# Wolbachia, Spiroplasma, and Rickettsia symbiotic bacteria in aphids (Aphidoidea)

**DOI:** 10.18699/VJ20.661

**Published:** 2020-10

**Authors:** D.A. Romanov, I.A. Zakharov, E.V. Shaikevich

**Affiliations:** Vavilov Institute of General Genetics of the Russian Academy of Sciences, Moscow, Russia Moscow Region State University, Mytishi, Moscow region, Russia; Vavilov Institute of General Genetics of the Russian Academy of Sciences, Moscow, Russia; Vavilov Institute of General Genetics of the Russian Academy of Sciences, Moscow, Russia Martsinovsky Institute of Medical Parasitology, Tropical and Vector Borne Diseases, Sechenov First Moscow State Меdical University, Moscow, Russia

**Keywords:** aphids, endosymbionts, PCR, plant pests, mutualism, тля, эндосимбионты, ПЦР, вредители растений, мутуализм

## Abstract

Aphids are a diverse family of crop pests. Aphids formed a complex relationship with intracellular bacteria. Depending on the region of study, the species composition of both aphids and their facultative endosymbionts varies. The aim of the work was to determine the occurrence and genetic diversity of Wolbachia, Spiroplasma and Rickettsia symbionts in aphids collected in 2018–2019 in Moscow. For these purposes, 578 aphids from 32 collection sites were tested by PCR using specific primers. At least 21 species of aphids from 14 genera and four families were identified by barcoding method, of which 11 species were infected with endosymbionts. Rickettsia was found in six species, Wolbachia in two species, Spiroplasma in one species. The presence of Rickettsia in Impatientinum asiaticum, Myzus cerasi, Hyalopterus pruni, Eucallipterus tiliae, Chaitophorus tremulae and Wolbachia in Aphis pomi and C. tremulae has been described for the first time. A double infection with Rickettsia and Spiroplasma was detected in a half of pea aphid (Acyrthosiphon pisum) individuals. For the first time was found that six species of aphids are infected with Rickettsia that are genetically different from previously known. It was first discovered that A. pomi is infected with two Wolbachia strains, one of which belongs to supergroup B and is genetically close to Wolbachia from C. tremulae. The second Wolbachia strain from A. pomi belongs to the supergroup M, recently described in aphid species. Spiroplasma, which we observed in A. pisum, is genetically close to male killing Spiroplasma from aphids, ladybirds and moths. Both maternal inheritance and horizontal transmission are the pathways for the distribution of facultative endosymbiotic bacteria in aphids.

## Abstract (ru)

Тли – разнообразное семейство вредителей сельскохозяйственных культур. Тли сформировали сложную взаимосвязь с внутриклеточными бактериями, известными как эндосимбионты, которые оказывают как положительное, так и отрицательное влияние на хозяина, что может иметь практическое значение. В раз- ных регионах мира состав факультативных симбионтов в популяциях тлей варьирует. Задачей работы было установить распространение и генетическое разнообразие симбионтов Wolbachia, Spiroplasma и Rickettsia в тлях, собранных в 2018–2019 гг. в Москве и Подмосковье. Для этого 578 тлей из 32 мест сбора тестировали методом ПЦР, используя специфические праймеры для мтДНК тлей, Wolbachia, Spiroplasma и Rickettsia. Мето- дом молекулярно-генетического анализа определено не менее 21 вида тлей из 14 родов и четырех семейств. Одиннадцать видов оказались инфицированы эндосимбионтами, а именно: у шести видов обнаружены Rickettsia, у двух видов – Wolbachia, у одного – Spiroplasma. Впервые выявлено заражение бактерией Rickettsia у Impatientinum asiaticum, Myzus cerasi, Hyalopterus pruni, Eucallipterus tiliae, Chaitophorus tremulae и бактерией Wolbachia у Aphis pomi и C. tremulae. У половины особей гороховой тли Acyrthosiphon pisum установлено двой- ное заражение Rickettsia и Spiroplasma. Впервые выявлены риккетсии у шести видов тлей, которые генетиче- ски отличаются от известных ранее. Впервые обнаружено заражение яблонной тли A. pomi двумя штаммами Wolbachia, причем один из штаммов относится к супергруппе В и генетически близок с Wolbachia из осино- вой тли C. tremulae, а второй штамм относится к супергруппе М, недавно описанной у видов тлей. Spiroplasma, найденная нами у A. pisum, генетически близка Spiroplasma, вызывающей андроцид у тлей, божьих коровок и молей, и кластеризуется с S. ixodetis. Разнообразие ДНК симбионтов убедительно свидетельствует о том, что как материнское наследование, так и горизонтальный перенос являются путями распространения факульта- тивных бактерий у тлей.

## Introduction

Aphids (Aphidoidea) are an insect superfamily from the order Hemiptera, which includes about 10 families or subfamilies and about 5000 species. Aphids are widespread sap-feeding plant pests and can transmit at least 30 % of plant viruses (Augustinos et al., 2011). A complex interplay with intracellular bacteria also known as endosymbionts is commonly observed in aphids. Obligate associations with Buchnera aphidicola in aphids provide insect hosts with essential amino acids absent in sap (Douglas, 1998). Apart from that, there are nine species of facultative symbionts of aphids (see the review by Guo et al., 2017) coexisting with Buchnera, having both positive and negative effects on the hosts.

Depending on the species, facultative intracellular symbiotic bacteria may increase the resistance of aphids against heat stress, parasitoid wasps, and fungal infections, participate in the synthesis of essential nutrients for the host with the obligate symbiont, and facilitate the interaction of aphids with plants that they feed on (Guo et al., 2017). However, it has been shown that the Rickettsia bacteria have a negative effect on the fitness of the pea aphid Acyrthosiphon pisum and suppress the activity of Buchnera aphidicola (Sakurai et al., 2005). Spiroplasma bacteria in A. pisum shorten the life span of aphids and inhibit their reproduction (Simon et al., 2007, 2011), albeit have a (minor) protective effect against the parasitoid wasp Aphidius ervi (Mathé‐Hubert et al., 2019). The role of Wolbachia in aphids is not yet fully understood (De Clerck et al., 2015; Manzano-Marín, 2019), but Wolbachia endosymbionts in the Asian citrus psyllid Diaphorina citri repress holin promoter activity in the alpha-proteobacteria ‘Candidatus Liberibacter asiaticus’, a citrus disease agent, thereby killing the bacteria and preventing the disease from spreading (Jain et al., 2017).

The present paper is dedicated to studying facultative endosymbionts Wolbachia, Spiroplasma, and Rickettsia in aphids. Wolbachia is the most common genus of symbiotic bacteria in insects, aphids not being an exception. Wolbachia infection has been recorded in 82 aphid species (Zytynska, Weisser, 2016). Two genera of symbionts, Rickettsia (Sakurai et al., 2005) and Spiroplasma (Fukatsu et al., 2001), were recorded in A. pisum. Both Spiroplasma and Rickettsia were recorded in the cowpea (Vigna sinensis) aphid Aphis craccivora (Brady et al., 2014) and the bean aphid Aphis fabae (Zytynska et al., 2016). Spiroplasma were also recorded in tropical aphids, specifically the citrus aphid Aphis citricidus and polyphagous Aphis aurantii (Guidolin, Cônsoli, 2018). Rickettsia were recorded in the blackberry aphid Amphorophora rubi (Haynes et al., 2003) and the cotton aphid Aphis gossypii (Jones et al., 2011). The symbiont composition varies in aphid populations around the world (Augustinos et al., 2011; Zytynska, Weisser, 2016; Guo et al., 2017; Guidolin, Cônsoli, 2018). For instance, Wolbachia, Spiroplasma, and Rickettsia were not recorded among facultative aphid symbionts in the Saratov Region, Russia (Malyshina et al., 2014).

Symbiont infection research in aphids is of practical value since such studies are instrumental in developing new strategies of controlling pathogen transmission in plants (Heck, 2018). Depending on the symbiont type, the information on infection may be used for symbiont elimination or transinfection with a specific strain of bacteria to inhibit the vector’s ability for pathogen transmission.

The goal of the present study was to investigate the incidence and genetic diversity of the Wolbachia, Spiroplasma, and Rickettsia symbionts in the samples of aphids collected in Moscow and the cities of Zvenigorod and Lyubertsy in the Moscow Region. Thus, 578 aphids from 32 collection sites were PCR-tested using specific primers for Wolbachia, Spiroplasma, and Rickettsia. The taxonomic positions of aphid hosts and their symbionts were identified based on the sequences of aphid and bacterial genes.

## Materials and methods

Aphids were collected in July–September 2018 and in May 2019 in Moscow, Zvenigorod, and Lyubertsy (Table 1). A total of 32 aphid samples were collected from 17 plant species. The collected adult aphids were preserved in 96 % ethanol.

**Table 1.**
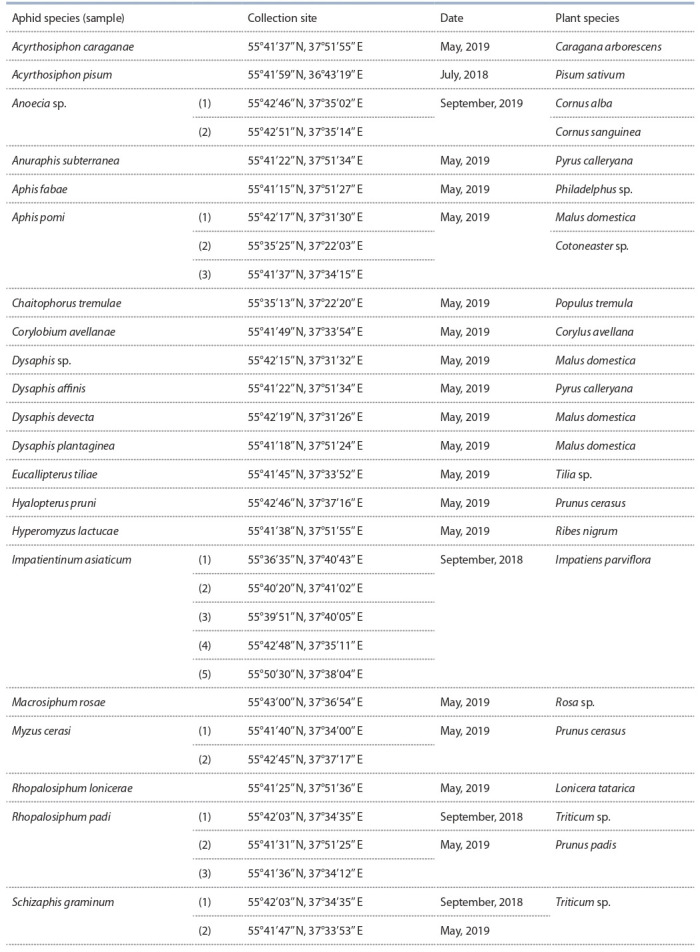
Aphid and plant species and their dates and sites of collection

Phenol-chloroform extraction was used to isolate total DNA from individual specimens (Sambrook et al., 1989). Amplification reactions were performed in a 25-μl volume using the universal Encyclo Plus PCR kit (Evrogen, Moscow) following the manufacturer’s protocol. All the reactions were performed using the MiniAmp Plus thermocycler (Applied Biosystems). Aphid species were identified based on PCR using universal primers LCO1490 and HCO2198 complementary to the 5′ end of the mitochondrial cytochrome c oxidase subunit I gene (COI ), as described earlier (Folmer et al., 1994).

Symbionts were identified using specific primers as follows: RicF141 and RicR548 for the Rickettsia gltA gene (Goryacheva et al., 2017), spi_f1 and spi_r3 for Spiroplasma 16S rRNA (Sanada-Morimura et al., 2013), ftsZ-F1 and ftsZ-R1 for the Wolbachia ftsZ gene (Baldo et al., 2006). The amplification procedure consisted of initial denaturation for 4 min 30 sec at 94 °C followed by 36 amplification cycles as follows: denaturation for 30 sec at 94 °C, annealing for 30 sec at 59 °C (53 °C for spi_f1 and spi_r3; and 56 °C for ftsZ ), and extension for 40 sec (1 min for spi_f1 and spi_r3) at 72 °C. The PCR was finalized by an extension for 5 min at 72 °C.

The PCR results were visualized by agarose gel electrophoresis (1.5 %). DNA fragments were eluted from gel using the DNA isolation kit for agarose gels Cleanup Mini (Evrogen, Moscow) under the manufacturer’s instructions. The purified PCR products were sequenced by Evrogen (Moscow). The newly acquired COI gene sequences were registered in the GenBank database under accession numbers МТ302332– МТ302357; gltA Rickettsia, under MT302358–MT302364; ftsZ Wolbachia, under MT302365–MT302368; and 16S Spiroplasma, under MT302369.

Sequence chromatograms were analyzed using the DNASTAR Lasergene 6 software (Clewley, 1995; Burland, 2000). The newly obtained sequences were compared to the previously available ones using the Barcode of Life (BOLD, http://www.barcodinglife.com/) and GenBank databases (https://blast.ncbi.nlm.nih.gov/Blast.cgi). Dendrograms were plotted using the neighbor-joining method, Kimura evolutionary model, and 1000 bootstrap replications in the MEGA 6.06 software (Tamura et al., 2013). DNA sequences in the dendrograms were provided with the GenBank and PubMLST (in the case of Wolbachia) registration numbers on the right from the isolate name. The divergence between nucleotide sequences was calculated based on p-distances (per-site differences) using the MEGA 6.06 software (Tamura et al., 2013).

## Results

**Diversity of aphid species**

**Fig. 1.**
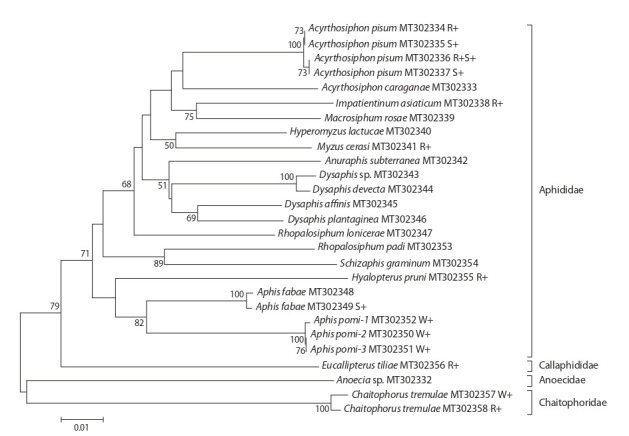
Phylogenetic reconstruction of the studied aphids species based on the analysis of the 630-bp nucleotide sequences of the mitochondrial COI gene. Individuals infected with Wolbachia, Rickettsia or Spiroplasma are designated W+, R+, S+, respectively.

To identify aphid species, the DNA barcoding method was used. The nucleotide sequences of mitochondrial COI gene were used to identify at least 21 aphid species in 32 samples, with 19 of them identified to a species level, and two, to a genus level, namely Dysaphis sp. from the apple tree Malus domestica and Anoecia sp. from the white dogwood Cornus alba and the common dogwood Cornus sanguinea. In latter cases, the sequence similarity was the highest with Dysaphis apiifolia (97 %) and Anoecia fulviabdominalis (96 %), respectively. The aphids collected represent four families as follows: Anoeciidae, Callaphididae, Chaitophoridae, and Aphididae (Fig. 1). Evolutionary divergence values amount to 6–16 % between the aphid genera, 0.8–6.6 % between the Dysaphis species and 6.3 % between Aphis species. Two mtDNA haplotypes were recorded in three species, namely Aphis pomi, Chaitophorus tremulae, and A. pisum (see Fig. 1). The differences between COI haplotypes were 0.2 % in A. pomi, 0.6 % in C. tremulae, and 0.16 % in A. pisum.

The apple aphid A. pomi, bird cherry-oat aphid Rhopalosiphum padi, black cherry aphid Myzus cerasi, and Asian balsam aphid Impatientinum asiaticum were found in two or more collection sites (see Table 1). Two aphid species were found in the same plant species in the following cases: Hyalopterus pruni and M. cerasi in cherry trees, A. pomi and Dysaphis devecta in apple trees, Anuraphis subterranea and Dysaphis affinis in pear trees, with the last aphid species coexisting on the leaves of the same tree (see Table 1). One aphid species was observed in different plant species in the following two cases: R. padi in bird cherry trees and wheat and A. pomi in apple trees and cotoneaster.

**Symbiotic bacterial infection**

The presence of Wolbachia, Spiroplasma, and Rickettsia was analysed in 578 specimens of 21 aphid species and the infections were detected in A. pisum (Spiroplasma and Rickettsia), I. asiaticum, M. cerasi, H. pruni, Eucallipterus tiliae (Rickettsia), A. pomi (Wolbachia), and Chaitophorus tremulae (Rickettsia and Wolbachia) (Table 2). The C. tremulae specimens infected with Rickettsia and Wolbachia had different mtDNA haplotypes (see Fig. 1).

**Table 2.**
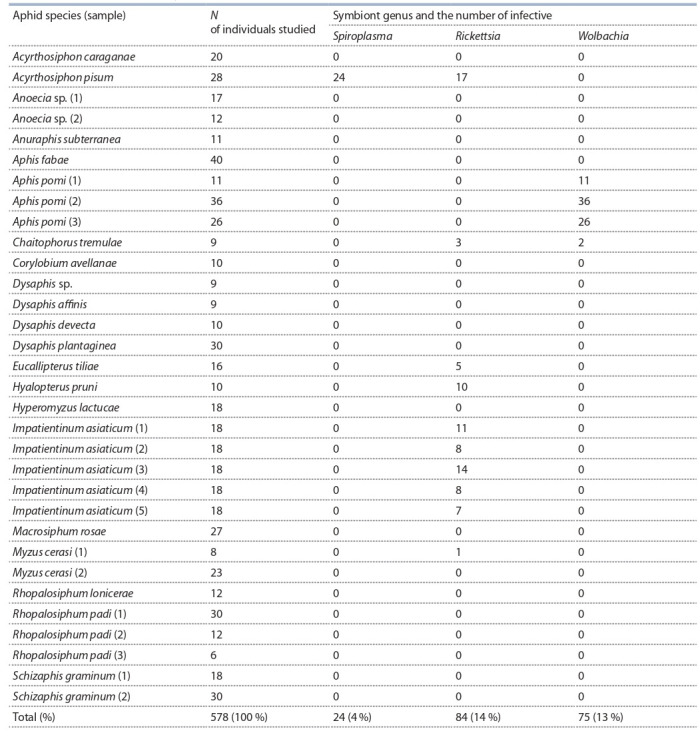
The occurrence of the symbionts in aphids

Rickettsia were found in 84 aphid specimens representing six species; Wolbachia, in 75 specimens of two species; and Spiroplasma, in 24 specimens of one species (see Table 2). Individual aphid specimens are typically infected by only one type of symbiotic bacteria out of three. However, Rickettsia/ Spiroplasma coinfection was recorded in A. pisum in 13 of 28 individual specimens. In adult A. pisum specimens, 100 % were infected with one or two types of endosymbionts. The presence of Rickettsia in I. asiaticum, M. cerasi, H. pruni, E. tiliae, and C. tremulae and Wolbachia in A. pomi and C. tremulae has been described for the first time.

**Fig. 2.**
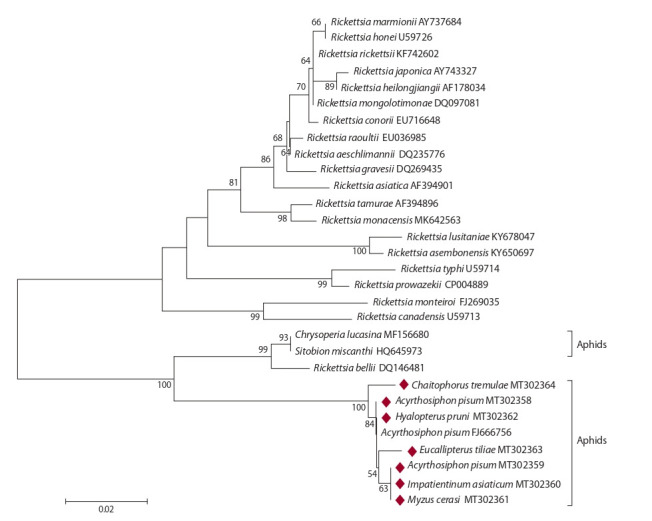
Phylogenetic reconstruction of Rickettsia diversity according to the analysis of 343 bp of the gltA gene. Bacteria from aphids are indicated by host species. The bacterial isolates obtained in this study are highlighted with diamonds.

**Bacteria of the genus Rickettsia.** Aphid Rickettsia were clustered with the R. bellii group (Fig. 2). Rickettsia in E. tiliae and C. tremulae had species-specific gltA alleles. Rickettsia in A. pisum had two alleles, gltA^1^ and gltA^2^ (GenBank entries MT302358 and MT302359), differing in one nucleotide substitution. The gltA^1^ allele was observed in one A. pisum specimen, and gltA^2^, in seven. The gltA^1^ allele was also observed in Rickettsia from H. pruni, and gltA^2^, in Rickettsia from I. asiaticum and M. cerasi. The gltA1 allele was identical to the Rickettsia DNA from A. pisum strain PAR (the USA) registered in GenBank as FJ666756 (see Fig. 2). The gltA2 allele has been discovered for the first time. The newly obtained Rickettsia DNA sequences differed significantly from bacterial DNA in Sitobion miscanthi aphids (HQ645973) genetically close to R. bellii (see Fig. 2). The evolutionary divergence value between the R. bellii group and Rickettsia group in the present paper amounted to 8.2 %, which greatly exceeded the divergence between, for example, the R. typhi and R. prowazekii species (2 %).

**Fig. 3.**
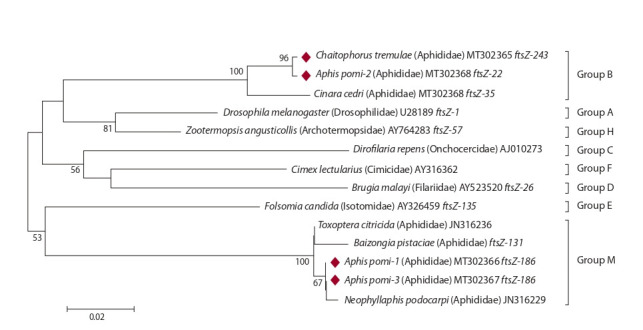
Phylogenetic reconstruction of Wolbachia diversity according to the analysis of 466 bp of the ftsZ gene. Wolbachia supergroups are shown on the right. Bacteria are indicated by host species. The bacterial isolates obtained in this study are highlighted with diamonds.

**Bacteria of the genus Wolbachia.** Within the genus Wolbachia, there are 16 phylogenetic supergroups (Glowska et al., 2016). Wolbachia from Aphis pomi #1 (MT302366) and #3 (MT302367) were clustered with supergroup М bacteria, and Wolbachia from Chaitophorus tremulae (MT302365) and A. pomi #2 (MT302368), with supergroup В (Fig. 3). The DNA differences in the ftsZ gene sequence of Wolbachia from A. pomi #2 (MT302368) and #1 (MT302366) amounted to 14.3 % (67 of 466 bp). At the same time, the ftsZ gene allele of Wolbachia from A. pomi #2 (MT302368) had only three different substitutions (0.6 %, 3 from 469 bp) if compared to the Wolbachia DNA from the Aspen leaf aphid Chaitophorus tremulae (MT302365).

The sample of A. pomi infected with group B Wolbachia was collected from cotoneaster, and the samples of A. pomi infected with group M Wolbachia, from cotoneaster and apple trees (see Table 1). The distance between the collection sites, where A. pomi were collected from the same plant species but infected with different Wolbachia strains, was over 20 km. At the same time, the distance between the collection sites, where A. pomi were collected from different plant species but infected with the same Wolbachia strain, did not exceed 4 km (see Table 1).

**Fig. 4.**
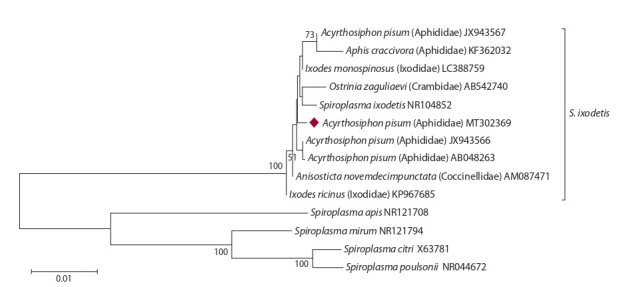
Phylogenetic reconstruction of Spiroplasma diversity according to the analysis of 974 bp of the 16S rRNA gene. Insect bacteria are indicated by host species. The bacterial isolate obtained in this study is highlighted with a diamond.

**Bacteria of the genus Spiroplasma.** Spiroplasma observed in A. pisum were clustered with the bacteria encountered in A. pisum in Japan (AB048263), Britain (JX943566, JX943567), and A. craccivora from the USA (KF362032) (Fig. 4). Variation of 16S rRNA genes of Spiroplasma in aphids from A. pisum collected in geographically separated sites amounted to 0.3–0.6 % (5–8 nucleotide substitutions per 974 bp). All of them are included in the Spiroplasma ixodetis clade. The latter also had the symbionts of other insects, such as lady beetles Anisosticta novemdecimpunctata (АМ087471) and the moth Ostrinia zaguliaevi (АВ542740). Nucleotide variation within this clade did not exceed 0.6 %. However, nucleotide variation between the clades of the genus Spiroplasma reached 10–16 %.

## Discussion

The study has demonstrated that at least 21 aphid species were represented in the 32 samples investigated. Most aphid species studied (18 of 21) belong to the family Aphididae, while the families Anoeciidae, Callaphididae, and Chaitophoridae are each represented by one species. Two species were not confined to their plant hosts, whereas 19 aphid species were encountered on particular plant species.

For the first time, Rickettsia infection has been observed in I. asiaticum, M. cerasi, H. pruni, E. tiliae, and C. tremulae, and Wolbachia infection, in A. pomi and C. tremulae. As opposed to other studies (see Zytynska, Weisser, 2016), aphid species collected in Moscow in the present research were mostly infected by Rickettsia rather than Wolbachia. The specimens of the Aspen leaf aphid C. tremulae infected with Rickettsia and Wolbachia have different mtDNA haplotypes, although it has yet to be studied in a larger number of samples whether cytoplasmic components are inherited together in this particular aphid species. Rickettsia and Spiroplasma coinfection was recorded in half of the specimens of the green pea aphid A. pisum. Many authors have already noted the presence of Rickettsia and Spiroplasma among the A. pisum facultative symbionts in Europe (Nyabuga et al., 2010; Ferrari et al., 2012; Gauthier et al., 2015), the USA (Russell et al., 2013; Smith et al., 2015), and Japan (Tsuchida et al., 2002). Among 28 A. pisum specimens in the sample from Zvenigorod, infection rates were 61 % (Rickettsia) and 86 % (Spiroplasma), which is several times as large as the previous figures, 8 and 3 % in 119 isofemale lines from 81 populations in Japan (Tsuchida et al., 2002), 48 and 9 % among 318 specimens in the USA (Russell et al., 2013), 4 and 22 % in 30 lines (Nyabuga et al., 2010), 23 and 27 % in samples collected from eight legume species from England and Germany (Ferrari et al., 2012). It is possible that these high bacterial infection rates only existed for a short period, since both Spiroplasma and Rickettsia reduce the life span of aphids and inhibit their fertility (Simon et al., 2007, 2011; Mathé‐Hubert et al., 2019). According to our observations, prolonged laboratory cultivation of A. pisum previously resulted in a loss of Spiroplasma symbiotic bacteria.

Aphid Rickettsia in our collections form a separate cluster. It was shown earlier that Rickettsia from A. pisum were attributed to the R. bellii group based on a comparison of four bacterial genes (Weinert et al., 2009). Other bacterial genes in different aphid species are to be studied to find out whether they form a separate species within the genus Rickettsia. The bacterial group R. bellii is the basal group of Rickettsia formed before the pathogenic spotted fever group and murine typhus group (Stothard et al., 1994). Four genetically different alleles of the Rickettsia gltA gene were discovered in the six aphid species studied, two of them encountered in A. pisum. One allele (MT302359) was observed in specimens from three aphid genera, another one (MT302358), in two genera, and the two remaining species had unique allelic variants of symbionts (MT302363-64). Thus, we may assume that different aphid species were infected with Rickettsia independently.

Spiroplasma discovered in A. pisum based on DNA analysis of the 16S rRNA gene is clustered with the bacteria previously observed in A. pisum and A. craccivora. It has been shown that Spiroplasma in A. pisum cause male offspring deaths at early larval stages, i. e. androcide or male-killing (Simon et al., 2011), and are genetically close to the Spiroplasma that caused androcide in aphid predators, i. e. lady beetles (Tinsley, Majerus, 2006) and moths (Tabata et al., 2011). All these bacteria are a part of the Spiroplasma ixodetis group. S. ixodetis is an endosymbiont described in ticks, but it is also common in other arthropods. Phylogenetic studies have shown that S. ixodetis strains are subjected to multiple horizontal transfers between ticks and other arthropods including aphids (Binetruy et al., 2019).

The present study has discovered Wolbachia infection in the apple aphid A. pomi for the first time. Wolbachia was found in 100 % of A. pomi specimens in our collections, even though this symbiont had not been observed in this aphid species from Greece earlier (Augustinos et al., 2011). The apple aphid A. pomi from three collection sites in Moscow (#1, 2, and 3 in Table 1) were infected with two Wolbachia strains, one attributed to supergroup В and another, to supergroup М (see Fig. 3), which had been described as prevalent in the aphid species of Spain, Portugal, Greece, Israel, and Iran (Augustinos et al., 2011), China (Wang et al., 2014), and the Azores (Moreira et al., 2019). Since the data shows the group’s low genetic variation, one might assume that it emerged quite recently and spread rapidly across aphid populations (Wang et al., 2014).

The results allow us to assume that infection of A. pomi with a specific Wolbachia strain is not linked to the plant host spespecies, but rather to its habitat. The noteworthy fact of discovering allelic variants of the Wolbachia ftsZ gene with only slight differences in various aphid species (A. pomi and C. tremulae) may imply the possibility of bacterial gene exchange during horizontal transfers of Wolbachia among insects, for example, through parasites or plants. This hypothesis is corroborated by the discovery of closely related alleles of Wolbachia genes in other unrelated insects (Ilinsky, Kosterin, 2017; Shaikevich et al., 2019). Symbiont DNA diversity is persuasive evidence of maternal inheritance and horizontal transfer being the key distribution mechanisms of facultative bacteria in aphids.

## Conclusion

While laboratory research results provide an increasingly better understanding of the role of aphid symbionts, data on symbiont distribution in aphid populations in natural ecosystems are still insufficient. The present paper provides the first report on the genetic diversity of bacterial endosymbionts in previously understudied aphid species. Aphid population screening in Moscow and the Moscow Region made it possible to newly identify Rickettsia infection genetically different from infection with R. bellii, to which aphid symbionts are typically attributed, in six aphid species (Weinert et al., 2009). Whether these bacteria should be considered a new species is to be decided by studying a larger number of Rickettsia alleles. An apple aphid A. pomi infection with two Wolbachia strains has been discovered for the first time, one being in the supergroup B strain, which is genetically close to Wolbachia from Aspen leaf aphids. The second Wolbachia strain from A. pomi belongs to supergroup М. Regardless of the strain, 100 % of A. pomi specimens in the present study were infected with Wolbachia, and we thus may assume that there is a selection mechanism of infected specimens in place, which involves the reproductive manipulations discovered in Spiroplasma from A. pisum (Simon et al., 2011). However, most aphid generations are asexual, and if symbiont infections are maintained in natural aphid populations by reproductive effects, then this reproductive phenotype has to be of great value for the insect host. The obvious advantage of androcide is that it prevents inbreeding in the aphid population (Simon et al., 2011). As for now, it is unclear whether these advantages outweigh the possible side effects of symbiont infection during the asexual phase of the lifecycle. The positive effect, i. e. resistance against natural enemies (parasitoid wasps, pathogenic fungi, and heat stress), seems to be the key driver of an increasing incidence of Wolbachia, Spiroplasma, and Rickettsia facultative endosymbionts in aphid populations.

## Conflict of interest

The authors declare no conflict of interest.
